# A Retrospective, Single-Institution Experience of Bullous Pemphigoid as an Adverse Effect of Immune Checkpoint Inhibitors

**DOI:** 10.3390/cancers14215451

**Published:** 2022-11-05

**Authors:** Walid Shalata, Sarah Weissmann, Sapir Itzhaki Gabay, Kim Sheva, Omar Abu Saleh, Ashraf Abu Jama, Alexander Yakobson, Keren Rouvinov

**Affiliations:** 1 The Legacy Heritage Center & Dr. Larry Norton Institute, Soroka Medical Center and Ben Gurion University, Beer Sheva 84105, Israel; 2 Medical School for International Health, Ben Gurion University of the Negev, Beer Sheva 84105, Israel; 3 Department of Dermatology, Soroka Medical Center and Ben Gurion University, Beer Sheva 84105, Israel; 4 Department of Dermatology and Venereology, Emek Medical Centre, Afula 18341, Israel

**Keywords:** bullous pemphigoid, pembrolizumab, nivolumab, immune checkpoint inhibitors, PD-L1 inhibitor, PD-1 inhibitor

## Abstract

**Simple Summary:**

This article investigates the cutaneous adverse immune effects induced by immune checkpoint inhibitors. These drugs target proteins expressed on cancer cells that aid in the avoidance of immune system detection and destruction. Immune checkpoint inhibitors inadvertently cause other immune-mediated adverse effects. Cutaneous toxicities are the commonest adverse effect from immunotherapy; furthermore, they are usually developed early in the course of treatment. A rare and severe cutaneous adverse event is Bullous Pemphigoid. This article investigates the average and median onset of these drug toxicities, as well as treatments. We found these side effects to be negatively skewed, indicating most cases occur several months into treatment.

**Abstract:**

Immune checkpoint inhibitors are a class of cancer treatment drugs that stimulate the immune system’s ability to fight tumor cells. These drugs are monoclonal antibodies targeting im-mune-inhibiting proteins on cancer cells, such as CTLA-4 and PD-1/PD-L1. Immune checkpoint inhibitors cause many immune-related adverse events. Cutaneous toxicities are of the most common adverse effects and occur with a range of severity. Bullous Pemphigoid is a rare adverse event with a high impact on quality of life that may occur after immune checkpoint inhibitor treatment. In this article, we investigate current research on immune checkpoint inhibitors, cutaneous adverse events, and common presentations and treatments, with a specific focus on Bullous Pemphigoid, its characteristics, onset timing, and treatment. Significant findings include a negative skew in the onset of presentation. Furthermore, we describe exclusive cases.

## 1. Introduction

Over the past several years, immune checkpoint inhibitors (ICIs) have become the cornerstone of cancer treatment. The immune system protects the body from foreign invaders, including cancer cells, by recognizing neoantigens or mutated proteins on tumor cells and facilitating their destruction. Tumor cells, however, express signals that inhibit the immune system, preventing their detection and destruction [1]. Immune checkpoint inhibitors block these inhibitory signals, promoting the immune system’s response. There are currently several different ICIs approved by the FDA, the first having been approved in 2011. Approved ICIs include monoclonal antibodies against programmed death-1 (PD-1), such as nivolumab or pembrolizumab [2]; ligands of PD-1 (PD-L1), such as atezolizumab; and cytotoxic T-lymphocyte-associated antigen-4 (CTLA-4), such as ipilimumab. These ICIs promote the immune system in different ways that complement each other, and ICIs are therefore often used in combination.

CTLA-4 is an activated T cell and regulatory T cell marker that binds to CD80 and CD86 on antigen-presenting cells with higher affinity than the stimulatory molecule B7, thereby inhibiting its activity and proliferation [3]. Ipilimumab blocks CTLA-4, the T cell-inhibiting signal that prevents immune cell detection of tumor cells. This allows T cells to recognize and fight tumor cells more effectively. Similarly, PD-L1 inhibits the immune system’s detection of tumor cells. PD-L1 is expressed on antigen-presenting cells and tis-sues such as the heart, muscle, and lung [4]. PD-L1 binds to PD-1 on T cells, causing the inhibition of the T cell response, proliferation, and survival, effectively preventing autoimmune reactions. PD-L1 and PD-1 interactions also inhibit the proliferation of regulatory T cells, which can protect against autoimmunity as well [4]. Tumor cells can express PD-L1, inhibiting T cells and thereby avoiding detection [4]. Blocking the PD-1/PD-L1 interaction with nivolumab, pembrolizumab, or atezolizumab thus promotes the immune response and detection of tumor cells. 

Since ICIs non-specifically activate T cells in the body, many different adverse effects arise. Typically, 70–90% of patients treated with ICIs experience immune-related adverse events (irAEs) and 10–15% of patients experience severe irAEs [5]. Fatalities from irAEs have been seen in up to 1.3% of patients [6]. irAEs most commonly occur within 1 year of treatment, but the risk of developing irAEs increases with time [7]. irAEs can result from autoimmune reactions due to the uninhibition of self-reactive T cells or attacks on homologous antigens to tumor cells in the body [8]. ICI toxicity usually presents as a skin rash, hepatitis, thyroiditis, hypohysitis, myocarditis, pericarditis, arthritis, colitis, uveitis, or pneumonitis [9,10].

Dermatotoxicities, or cutaneous toxicities, are of the most common irAEs, occurring in up to 30–50% of patients treated with ICIs [11]. The most common dermatotoxicities seen in patients include pruritis, exanthems, vitiligo, and lichenoid reactions [12]. Other less common dermatotoxicities may arise as immune-related adverse events, such as Bullous Pemphigoid (BP) and severe cutaneous adverse reactions (SCARS), including Stevens–Johnson Syndrome/Toxic Epidermal Necrolysis (SJS/TEN), Acute Generalized Exanthematous Pustulosis (AGEP), and Drug Reaction with Eosinophilia and Systemic Syndromes (DRESS).

BP is a rare autoimmune skin disorder that presents with blisters, urticarial lesions, and pruritis. BP as an immune-related adverse event is rare, occurring in approximately 1% of patients treated with anti-PD-1/PD-L1 therapy [13]. BP is associated with significant morbidity and mortality and the burden of the disease is often underestimated [13]. Although there have been numerous reviews on dermotoxicity and ICIs, there is a paucity of data on the timing of onset of dermatotoxicities, specifically Bullous Pemphigoid. In this study, we review immune checkpoint inhibitors, the dermatotoxicities they can cause, the incidence and timing of Bullous Pemphigoid, and the proposed treatments. We investigate the differences in timing of BP onset following different immune checkpoint inhibitors and cancers through a quantitative analysis.

## 2. Materials and Methods

This was a single-institution, retrospective, observational study without intervention. Ethical review and approval were waived for these cases by the Institutional Review Board of Soroka University Medical Center because all three cases did not involve any intervention or procedures specifically incurred for the purpose of human research. 

The study included patients who were admitted to Soroka Medical Center between January 2017 and March 2022. 

The inclusion criteria for the study were:

Patients aged 18 years or older.

Patients diagnosed with brain pineoblastoma, renal cell carcinoma, and urothelial carcinoma (advanced or metastatic diseases).

Patients treated only at Soroka Medical Center or who have a full follow-up history in Soroka Medical Center’s records.

Each patient admitted to Soroka Medical Center Oncology Institute is presented and discussed in a multidisciplinary team as required. This team includes a general medical and radiation oncologist, imaging and nuclear physician, pulmonologist, pathologist, and thoracic surgeon. The discussion is based on the patients’ status, pathology, and imaging. Each patient is assigned a primary physician who is responsible for the treatment course.

Patients with advanced or metastatic diagnoses are treated mainly by medical oncologists, and the treatment plan is based in general on the NCCN recommendations. Routine molecular profiling is performed for each patient when possible. 

**Figure 1:**
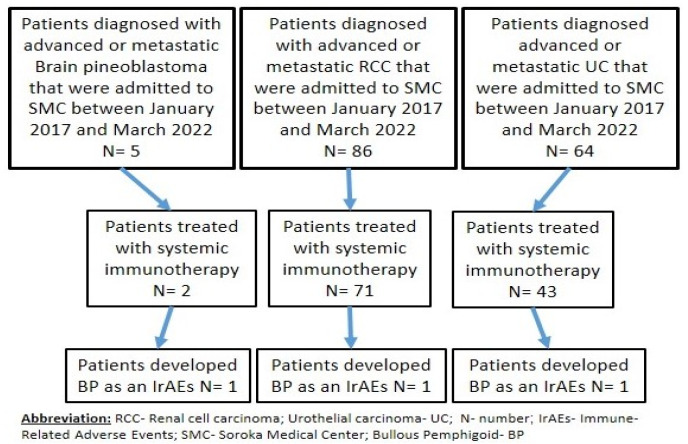
Flow diagram of the single-center, retrospective, observational study of advanced or metastatic brain pineoblastoma, renal cell carcinoma, and urothelial carcinoma.

We identified a total of three patients with advanced or metastatic brain pineoblastoma, renal cell carcinoma, and urothelial carcinoma that were treated with immunotherapy (Figure 1). 

The studies used in this article were retrieved from PubMed and published between 1 May 2015 and 1 October 2022, in order to obtain the most relevant and recent results. The search was limited to studies published over the past 6 years with the following search terms used: Bullous pemphigoid and ICIs, Dermatoxocities and ICIs, Cutaneous toxicities and ICIs. The search terms were chosen to include the broadest range of published papers on this topic. We focused on Bullous Pemphigoid specifically during the search in order to achieve better results. We reviewed all search results and included 55 relevant case studies and reviews. 

Each case report was appended to a table specifying their year, gender, age, immunotherapy, tumor, presentation, grade, time until onset, and treatment (Appendix A). The majority of case reports were published between 2018 and 2021. Mean and median time until onsets were calculated, and trends were analyzed qualitatively through a table and graph. In addition, we describe three new cases seen in patients admitted to Soroka Medical Center between January 2017 and March 2022, two of which have not been published before (with regard to treatment or diagnosis). Ethical review and approval were waived for these cases by the Institutional Review Board of Soroka University Medical Center because all three cases did not involve any intervention or procedures specifically incurred for the purpose of human research.

## 3. Exclusive Case Series

### 3.1. Case 1—Bullous Pemphigoid Induced by Nivolumab in Patient with Brain Pineoblastoma

The patient is a 47-year-old male with a history of brain pineoblastoma that was treated with craniotomy in 2016, which was followed up with radiotherapy and chemotherapy (75 mg/m^2^ of cisplatin every three weeks for two cycles). He has no family history of cancer, and his past medical history is significant only for hypercholesteremia (on simvastatin).

The patient remained in follow-up until January 2019, when he underwent a brain MRI that showed further disease progression. The patient was subsequently started on treatment with 240 mg of nivolumab every two weeks for the first month and later increased to 480 mg every four weeks thereafter. In March 2019, he developed a rash with surrounding erythema and erosions and bloody serous in his peripheral extremities. He was seen by a dermatologist who began treatment with topical betamethasone two times per day, which showed a partial response. In November 2019, the patient was hospitalized because the rash developed blisters and became pruritic. He denied any recent history of illness, fever, night sweats, or viral disease. The complete blood count was normal, the blood chemistry showed elevated liver enzymes, and PCR varicella-zoster virus (VZV) and herpes simplex virus (HSV) testing were negative. A skin biopsy of the blisters confirmed a diagnosis of BP (Table 1).

Due to the suspicion that this was an adverse effect of the nivolumab administration, the medication was promptly stopped. He was started on 1 mg/kg of prednisone and reported no change in the first three days of treatment. The dosage was consequently increased to 2 mg/kg for one week, which showed improvement, so it was decreased to 1.5 mg/kg. The patient reported new blisters on this new dosage, so the treatment regimen was once again changed and increased to 2 mg/kg of prednisone. Lastly, azathioprine (Imuran) 100 mg tablets once daily were added and showed a good response.

### 3.2. Case 2—Bullous Pemphigoid Induced by Ipilimumab and Nivolumab in Patient with Metastatic Clear Cell Renal Cell Carcinoma (mRCC)

The patient is a 69-year-old male with history of type 2 diabetes mellitus (on Metformin), diabetic retinopathy, dyslipidemia (on atorvastatin), and hypertension (on valsartan and aspirin). He has a 30 pack-year history of smoking and a family history of two sisters with breast cancer.

Upon initial workup for his hypertension, he underwent an abdominal ultrasound (US) that showed a right renal mass. He then underwent abdominal CT that showed a 4.5 cm mass in the right kidney, as well as a mass in the head of the pancreas, which was suspected to be metastatic in origin. The patient was therefore referred to the oncology department, where a chest CT was performed, showing metastasis to the lung and bone (right 5th rib). A segmental resection of the left lung was carried out. The histology report confirmed the finding of mRCC. The pancreatic mass was biopsied and showed scanty material with blood elements. A repeat pancreatic biopsy was refused by the patient. 

The patient was ultimately diagnosed with stage 4 RCC (T1NXM1). He received systemic intravenous immunotherapy with a combination of 1 mg/kg ipilimumab and 3 mg/kg nivolumab every 21 days. After four cycles of combination treatment, the patient received only 3 mg/kg nivolumab every 14 days. After ten cycles, the patient presented with blisters on his scalp, chin, and both hands. He was seen by a dermatologist who recommended treatment with combination therapy consisting of prednisolone and gentamicin (aflumycin) twice a day for two weeks, showing only a partial response. Nivolumab was subsequently stopped, and the patient was referred to an immunologist who recommended the addition of corticosteroid therapy (prednisone 1 mg/kg for two weeks).

A diagnosis of BP caused by his immunotherapy treatment regimen was suspected, so the patient was sent for a diagnostic confirmatory biopsy. The skin biopsy confirmed BP (Table 1).

### 3.3. Case 3—Bullous Pemphigoid Induced by Pembrolizumab in Patient with Urothelial Carcinoma (UC)

The patient is a 69-year-old male with history of type 2 diabetes mellitus (on metformin), hypertension (on enalapril and aspirin), hypercholesterolemia (on simvastatin) and chronic kidney failure. He has a 25 pack-year smoking history and no family history of cancer.

In January 2017, he complained of hematuria and underwent an US of the urinary tract that showed a mass in the urinary bladder. CT urography confirmed a mass in the right lower anterior bladder wall with urethral meatus involvement. The patient underwent transurethral resection of the bladder mass. Histopathologic results showed high grade UC with tumor invasion into the muscularis propria and vascular invasion. The patient was admitted for radical cystectomy with ileal conduit, and histopathological results found poorly differentiated invasive UC.

The patient was started on adjuvant systemic intravenous chemotherapy consisting of 1000 mg/m^2^ of gemcitabine on days one and eight every 21 days, as well as carboplatin (area under curve-4) on day one every 21 days for six cycles. A total body CT performed in February 2018 showed a new nodule in the right lower lobe of the lung. The patient was started on a fixed dose of 200 mg pembrolizumab on day one every 21 days and ultimately received a total of 34 cycles of pembrolizumab. In October 2020, the patient presented with multiple skin blisters with surrounding erythema, peeling skin with erosions, and bloody serous scabs on both hands and feet. Pembrolizumab was stopped due to the suspicion that this was an immunotherapy-related adverse effect. Dermatological referral resulted in the diagnosis of BP (Table 1). The patient was started on dermovate cream (clobetasol proprionate) to be used twice daily for two weeks. Corticosteroid therapy was also initiated with 1 mg/kg of methylprednisolone once a day daily for five days. The patient’s blisters improved, and the dosage was changed to 1.5 mg/kg of prednisone once daily, after which the patient was discharged. Ten days later, there was marked improvement in his blisters, as well a decrease in both the skin peeling and the skin erosions on his hands and feet. The dosage of prednisone was decreased to 1 mg/kg once daily and the patient returned two weeks later showing almost complete resolution in his symptoms.

## 4. Immune Checkpoint Inhibitors

### 4.1. Ipilimumab

Ipilimumab is a fully human recombinant monoclonal antibody targeting CTLA-4 [14]. Ipilimumab is the only FDA-approved inhibitor of CTLA-4 [15]. It is currently used in treatment of numerous cancers, including melanomas, renal cell carcinomas, hepatocellular carcinomas, colorectal cancers, and non-small-cell lung cancers without any contraindications (Table 2) [16].

**Figure 2:**
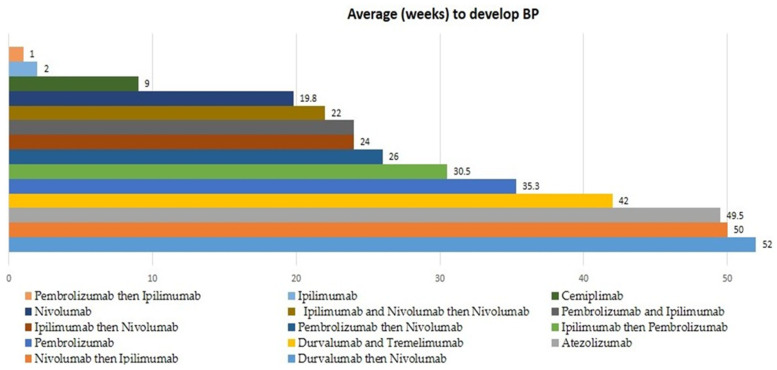
Median weeks until presentation of Bullous Pemphigoid adverse event by treatment type.

Immune-related adverse events develop in up to 90% of patients because of Ipilimumab’s ability to promote immune system activity [7]. The most common adverse effects of Ipilimumab include colitis, fatigue, diarrhea, pruritis, and rash. Severe immune-related reactions can be seen in less than 1% of patients presenting as enterocolitis, hepatitis, dermatitis, neuropathy, or endocrinopathy [16]. Cutaneous toxicities are the most common adverse effect of Ipilimumab, with 40–49% of patients having a rash or pruritus [17]. Patients usually present with maculopapular, erythematous rash, or pruritus within the first few doses. Of the patients who develop Bullous Pemphigoid, symptoms usually begin to appear 2 weeks after start of treatment. If Ipilimumab is combined with Nivolumab, symptoms present 24 weeks after start of treatment, and if it is combined with Pembrolizumab, symptoms begin after 30.5 weeks on average (Table 3 and Figure 2). Moisturizers may be recommended as a possible preventative measure for rash and pruritus due to their ability to improve skin hydration and provide a cooling effect from water evaporation, reducing itch symptoms [18,19,20]. Patients with other dermatologic adverse effects may be prescribed antipruritic medications or other topical steroids [18]. Discontinuation of Ipilimumab is recommended in cases of severe irAEs.

### 4.2. Pembrolizumab

Pembrolizumab is a humanized IgG4 monoclonal antibody targeting PD-1 [21]. Pembrolizumab is FDA-approved for treating melanoma, many types of squamous cell cancers, urothelial carcinomas, hepatocellular carcinoma, Merkel cell carcinoma, renal cell carcinoma (RCC), endometrial carcinoma, many different types of lymphomas, gastric and esophageal cancer, lung cancer, cervical cancer, triple-negative breast cancer, and other cancers that have high microsatellite instability, deficient mismatch repair, or a high tumor mutational burden (Table 2) [22].

Pembrolizumab causes immune-related adverse events in 70% of patients [7]. irAEs due to Pembrolizumab typically include fever, fatigue, cough, constipation, abdominal pain, diarrhea, decreased appetite, rash, pruritis, and musculoskeletal pain [22]. Approximately 42% of patients treated with Pembrolizumab develop dermatologic adverse effects, most commonly maculopapular eruption, pruritus, and hypopigmentation [23]. Patients who develop BP after Pembrolizumab typically present with symptoms 35.3 weeks after start of treatment. Patients who take Pembrolizumab after Ipilimumab may present with BP symptoms 30.5 weeks after treatment on average, and patients who take Pembrolizumab before treatment of Nivolumab or Ipilimumab present with symptoms around 25 weeks after treatment begins (Table 3 and Figure 2).

### 4.3. Nivolumab

Nivolumab is a human IgG4 monoclonal antibody targeting PD-1 [23]. It was approved by the FDA for treatment of melanoma, non-small-cell lung cancer, malignant pleural mesothelioma, classical Hodgkin lymphoma, colorectal cancer, RCC, squamous cell carcinoma of the head and neck, urothelial carcinoma, hepatocellular carcinoma, and esophageal squamous cell carcinoma (Table 2) [24].

Nivolumab causes immune-related adverse events such as pruritus, rash, diarrhea, colitis, hypothyroidism, hyperthyroidism, hypophysitis, hepatitis, and pneumonitis [25]. The most common irAEs found in patients treated with nivolumab include fatigue, rash, pruritis, and diarrhea [25]. Nivolumab causes cutaneous irAEs in up to 34% of patients [26]. Since Nivolumab inactivates PD-1 for up to 3 months, irAEs typically present some time after treatment is completed [27]. When treated with Nivolumab alone, patients who develop BP present with symptoms 19.8 weeks after beginning treatment. Patients who take Nivolumab after Durvalumab present with BP 52 weeks after treatment, while patients who take Nivolumab after Ipilimumab present with BP 24 weeks after treatment, and patients who take Pembrolizumab before Nivolumab present with BP, on average, 26 weeks before treatment (Table 3 and Figure 2). Patients with early incidence of rash and pyrexia had an enhanced tumor response and longer progression-free survival [28].

Ipilimumab and Nivolumab are commonly used in combination in the treatment of metastatic melanoma, advanced RCC, hepatocellular carcinoma, metastatic non-small-cell lung cancer, high microsatellite instability or mismatch repair-deficient metastatic colon cancer [16,29]. irAEs have higher incidence with this combination, causing up to 40% of patients to discontinue treatment [30].

### 4.4. Atezolizumab

Atezolizumab is a monoclonal humanized IgG antibody targeting PD-L1 [31]. It was approved by the FDA in 2017 and is used for the treatment of non-small-cell lung cancer, triple-negative breast cancer, hepatocellular carcinoma, melanoma, and urothelial carcinoma (Table 2) [32]. The most common adverse events occur in greater than 20% of patients [32]. Patients typically present with rash, alopecia, fatigue, nausea, vomiting, decreased appetite, constipation or diarrhea, cough, and dyspnea [32]. Atezolizumab can cause severe and even potentially fatal events, such as Acute Generalized Exanthematous Pustulosis (AGEP), Stevens–Johnson Syndrome (SJS), toxic epidermal necrolysis (TEN), and drug rash with eosinophilia and systemic symptoms (DRESS) [33]. On average, patients who develop BP take 49.5 weeks to develop symptoms from the onset of treatment (Table 3 and Figure 2). In cases of adverse events, it is recommended that Atezolizumab be withheld or permanently withdrawn [33].

### 4.5. Durvalumab

Durvalumab is a human monoclonal IgG antibody that blocks PD-L1 [34]. It is indicated in the treatment of non-small-cell lung cancer and platinum-containing therapy (Table 2) [35]. Patients may present with adverse effects such as rash, fatigue, nausea, constipation, urinary tract infections, edema, pneumonitis, endocrinopathies, nephritis, cough, and dyspnea [34]. More severe adverse effects such as hyperthyroidism or hypothyroidism, colitis, diarrhea, and hepatitis can be seen in patients and may even be fatal [36]. The most common adverse reactions in patients treated for urothelial carcinoma include fatigue, musculoskeletal pain, constipation, decreased appetite, nausea, peripheral edema, and urinary tract infections [34]. Durvalumab has been shown to induce dermatomyositis in rare cases [37]. Patients treated with Durvalumab and Nivolumab following presented with BP typically 52 weeks after initiation of treatment. Patients treated with Durvalumab and Tremelimumab presented with BP after an average of 42 weeks (Table 3 and Figure 2). Typical treatment of these adverse events includes pausing Durvalumab or decreasing the dose, but cessation of the drug may be required in cases with high-grade side effects [36].

### 4.6. Cemiplimab

Cemiplimab is a human monoclonal IgG antibody that inhibits PD-1 [38]. It is indicated in the treatment of cutaneous squamous cell carcinoma, basal cell carcinoma, and non-small-cell lung cancer (Table 2) [38]. Patients may present with immune-mediated adverse reactions, including pneumonitis, colitis, hepatitis, endocrinopathies, dermatologic adverse reactions, immune-mediated nephritis, renal dysfunction, and solid organ transplant rejection [38]. The most common adverse reactions are musculoskeletal pain, fatigue, rash, and diarrhea [38]. Dermatologic adverse reactions occurred in 1.6% of patients, with rash and dermatitis presenting most frequently [38]. Patients treated with Cemiplimab who develop BP typically present with symptoms around 9 weeks following the initiation of immune checkpoint inhibitors (Table 3 and Figure 2). Typical treatment of dermatologic adverse reactions includes corticosteroids or immunosuppressants, and in rare cases, cessation of Cemiplimab [38].

## 5. Dermatotoxicities

### 5.1. General

Dermatotoxicities are of the most common side effects of immune checkpoint inhibitors [39,40]. Dermatological irAEs may occur in up to 30–50% of patients treated with immune checkpoint inhibitors [40,41]. These effects typically present in a similar manner to primary dermatoses and may resemble autoimmune skin disorders [39,41,42,43]. The most common cutaneous adverse effects include pruritus, exanthems, vitiligo, and lichenoid reactions (Table 2). Patients are typically managed based on the severity of their symptoms, as determined by the Common Terminology Criteria for Adverse Events (CTCAE) [44]. Based on severity, topical or systemic steroids may be recommended, as well as temporary or permanent discontinuation of immune checkpoint inhibitors in specific cases [23].

Symptoms and cutaneous adverse effects present at different stages during treatment. Exanthems typically begin within the first few weeks of treatment and are often low-grade and self-limited [23]. Vitiligo usually begins several months into treatment and may follow an inflammatory phase [42,45]. Lichenoid dermatitis can present throughout treatment from weeks to months after beginning immune checkpoint inhibitors [39,46].

More severe cutaneous irAEs include immunobullous eruptions, which usually present as BP, acute generalized exanthematous pustulosis (AGEP), Drug reaction with Eosinophilia and Systemic Symptoms (DRESS), Drug-induced Hypersensitivity Syndrome (DIHS), Stevens–Johnson Syndrome/Toxic Epidermal Necrolysis (SJS/TEN), neutrophilic dermatoses such as Sweet’s syndrome, papulopustular and rosaceiform eruptions, cutaneous lymphoproliferative disorders, autoimmune connective tissue disorders such as lupus, dermatomysositis, and eosinophilic fasciitis, vasculitis, or alopecia areata (Table 4) [39,47,48].

Co-medications such as antibiotics, glucocorticoids, PPIs, NSAIDs, ACE inhibitors, metformin, and insulin are often prescribed with ICIs. Use of antibiotics, glucocorticoids >10 mg/day, PPIs, psychotropic drugs, morphine, and insulin at baseline is associated with decreased overall survival time and decreased tumor response [49]. Use of statins, ACEs and/or ARBs, NSAIDs, aspirin, and oral antidiabetic drugs is not associated with any change of outcomes. Co-medications also have a significant impact on irAEs, particularly cutaneous irEAs. Interestingly, co-medication at the onset of ICIs with antibiotics, glucocorticoids, PPIs, morphine, NSAIDs, aspirin, and psychotropic drugs is associated with a decreased occurrence of irAEs [49]. Previous studies have proposed that this finding is a result of decreased tumor response in the setting of co-medication use [49].

The mechanism of dermatotoxicity is thought to be due to immune checkpoint inhibitor-mediated overactivation of the immune response. Interestingly, patients with adverse effects such as vitiligo and lichenoid and spongiotic dermatitis have better outcomes of drug effectiveness and higher tumor responsiveness [50,51]. The difference in outcomes of patients with BP is still unclear, however. Nelson et al. presented data indicating BP may be related to more favorable responses to ICIs, while Faje et al. found that patients taking prednisolone over 7.5 mg/d have worse survival. [52,53].

### 5.2. Bullous Pemphigoid (Appendix A)

BP is a chronic autoimmune blistering disease commonly seen in patients over the age of 60. It is the most common autoimmune blistering disease and is defined by the presence of autoantibodies against hemidesmosomal proteins of the skin that lead to subepidermal separation and inflammation [54,55]. The autoantibodies may be tissue-bound or circulating and specifically target two main structural proteins: BP antigen 1 (termed BP230 antigen) and BP antigen 2 (termed BP180 antigen) [56,57]. BP typically presents with multiple clear blisters of varying sizes on erythematous skin, most commonly on the flexural surfaces of the extremities, lower parts of the abdomen, and in the groin area. Each bulla varies in size and can measure up to several centimeters in diameter [55]. Treatment for BP is dependent on the level of risk. Localized low-risk BP is treated with topical corticosteroids. For generalized disease, systemic treatment is usually achieved with prednisone, and can be supplemented with azathioprine, cyclophosphamide, or mycophenolic acid [56].

Blistering disorders are not commonly seen as an adverse effect in immune checkpoint inhibitor therapy [47]. Bullous pemphigoid, the most common blistering disorder, occurs in about 1% of patients treated with PD-1 or PD-L1 inhibitors [39,47,48]. BP can also be seen in patients treated sequentially with PD-1 and CTLA-4, but it is usually not found in patients treated with ipilimumab monotherapy [58,59]. BP as an immune checkpoint inhibitor adverse effect is often preceded by a non-bullous phase of pruritus and a nonspecific maculopapular rash. Patients may then present with blisters filled with serous or hemorrhagic fluid, either locally or generally, marking the onset of BP [60]. ICI-induced bullous pemphigoid, compared to idiopathic bullous pemphigoid, presents with a longer period of rash-free pruritus and more time between symptom onset and diagnosis [61].

The pathogenesis of BP in the setting of ICI treatment is still unclear. It is possible that ICIs cause a de novo induction of BP or that ICIs unmask a subclinical disease. BP is associated with higher age and neoplasia and therefore may be seen more commonly in these patients regardless of ICI use [62]. However, studies have found that patients with metastatic melanoma treated with BRAF inhibitors did not develop BP, suggesting BP may be induced by ICIs specifically [63,64]. Since data regarding hemidesmosomal autoantibodies in patients before ICI use are not available, it is difficult to determine the exact pathogenesis of ICI-induced BP.

BP in these settings is typically more severe and requires ICI treatment to be withheld or permanently discontinued while systemic steroids are initiated for treatment [65]. In mild cases, however, BP can be managed through conservative topical treatments or doxycycline/niacinamide [66]. For cases of BP that do not resolve with topical treatment or systemic steroids, rituximab may be indicated [67,68]. Though rituximab is a monoclonal antibody to CD20 on B cells, studies have shown that it may not interfere with the mechanisms of immune checkpoint inhibitors [67,68]. For BP with high IgE levels, data indicate that omalizumab may be effective [69]. Through these guidelines, physicians can meet the goals of continuing necessary ICI use while minimizing corticosteroid exposure in patients.

Diagnosis of BP as an immune checkpoint inhibitor adverse effect includes a dermatologic referral and skin biopsy. Biopsy of lesional and perilesional tissue allows for the identification of intraepidermal or subepidermal involvement by hematoxylin and eosin staining. The diagnosis can be confirmed by direct immunofluorescence (DIF), indirect immunofluorescence (IIF) using monkey esophagus, and ELISA serological testing for autoantibodies [60]. DIF confirming BP shows linear deposits of immunoglobulin G (IgG) and complement component 3 (C3) along the basement membrane. IIF confirming BP shows a band-like pattern along the basement membrane using monkey esophagus [60]. ELISA results show the detection of anti-BP230 or anti-BP180 antibodies [42]. Other subepidermal blistering disorders such as epidermolysis bullosa acquisita (EBA) must be ruled out before confirming a diagnosis of BP.

### 5.3. Treatment

Cutaneous immune reaction adverse events are classified by severity according to the Common Terminology Criteria for Adverse Events (CTCAE) [44]. Maculopapular rashes, the most commonly occurring cutaneous adverse event, can be categorized into four grades according to the percentage of body surface area (BSA) affected, symptoms (e.g., pruritus, burning, tightness), and limitation of self-care or activities of daily life (ADL) [44]. Grade 1 includes macules/papules covering <10% BSA with or without symptoms of pruritus, burning, or tightness; Grade 2 includes macules/papules covering 10%-30% BSA with or without symptoms and with ADL limitations; Grade 3 includes macules/papules covering >30% BSA with or without symptoms and limiting self-care ADL; and Grade 4 includes a papulopustular rash associated with life-threatening superinfections, SJS, TEN, and bullous dermatitis covering >30% BSA and requiring intensive care unit admission [44].

Most Grade 1 dermatologic events are treated symptomatically with topical emollients, oral antihistamines, and/or mild-strength topical corticosteroids [44]. Immune checkpoint inhibitors do not need to be discontinued. Grade 2 cutaneous events may be treated with topical emollients, oral antihistamines, and medium or high-strength topical steroids [44]. Immune checkpoint inhibitors do not need to be immediately discontinued, but the patient should be monitored weekly for improvement, and if the cutaneous event does not improve to Grade 1, ICIs should be discontinued [44]. Grade 3 dermatologic events require immediate discontinuation of ICIs until the dermatotoxicity resolves to Grade 1. These adverse events should also be treated with topical emollients and oral antihistamines, and high-strength topical steroids and systemic corticosteroids may be added, depending on severity [44]. Grade 4 adverse cutaneous events such as SJS, TEN, and high-grade bullous dermatitis require immediate interruption of ICIs, and the patient should be admitted to the intensive care unit for close dermatological monitoring [44]. The treatment of these patients typically includes intravenous prednisolone that may be tapered with symptom resolution [44].

## 6. Discussion

Dermatotoxicities are the most common side effect of immune checkpoint inhibitors. BP is a rare adverse event that causes considerable morbidity and mortality. Since its presentation is more severe as an adverse drug event, it is important for physicians and health practitioners to know how it typically presents and the timing of average onset.

There is a significant amount of variation in the time between ICI treatment and BP presentation based on the immunotherapy used. BP presents 1–2 weeks after Ipilimumab following Pembrolizumab and Ipilimumab, respectively, while Pembrolizumab, Druvalumab, Tremelimumab, and Ipilimumab after Nivolumab, and Nivolumab after Durvalumab are associated with BP almost a year after treatment begins. The average number of weeks between onset of BP and the start of ICIs can be seen in Table 2 and Table 3. The bell curve of weeks until presentation of BP is negatively skewed, demonstrating that, in the majority of cases and treatments, BP presents many weeks after start of treatment, but there are a few cases with BP presenting as soon as a couple of weeks after the initiation of immunotherapy treatment. Given this information, physicians and health care providers should be aware of BP as a potential adverse effect not just weeks after starting treatment, but several months after as well.

Ipilimumab after Pembrolizumab and Ipilimumab alone are the treatments that present with BP the earliest (within 1–2 weeks). Nivolumab after Ipilimumab and Nivolumab, Pembrolizumab, and Ipilimumab, Nivolumab after Ipilimumab, and Nivolumab after pembrolizumab typically present with BP after 6 months. The treatments that take the longest for BP to present are Ipilimumab after Nivolumab, and Nivolumab after Durvalumab and Atezolizumab. It is unclear why different therapies cause BP during different time intervals.

Although the pathogenesis of BP as an ICI adverse event is also unclear, it is thought to be due to a depletion of CD4+, CD25+, and Foxp3+ regulatory T cells. This decrease in T regulatory cells may allow for the proliferation of autoantibody-secreting B cell clones targeting antigens in the basement membrane of the skin [70]. PD-1/PD-L1 blockers cause reactivation of exhausted T cells, which may allow for interactions between PD-1/PD-L1-expressing B cells and PD-1+ follicular helper T cells. This would then cause a primarily humoral B cell germinal center response [71,72]. Future research should investigate the mechanisms behind BP as an immune-related adverse event.

There is also currently a lack of understanding as to why some patients develop BP as an immune-related adverse event, while others do not. Some studies suggest that it is related to having a common antigen on both cancer cells and in the dermoepidermal junction [47]. BP180 is an antigen that has been found on tumor cells, as well as NSCLC cells and the basement membrane of the skin [73]. It is possible that BP arises as a result of overactive T cells targeting both BP180 on tumor cells and BP180 in the basement membrane. BP180 can be found in other tissues as well, and other adverse events may also occur through this mechanism [74]. As previously mentioned, several treatments may lead to an increased incidence of BP. Additionally, there has been an increase in reported cases of diabetic patients associated with BP in the last decade during treatment with gliptins, which are used to treat type 2 diabetes mellitus (T2DM). The second and third cases in our study, however, received metformin for T2DM [75]. Although Chouchane et al. suspected an association between metformin and BP, it was concluded that there is no significant association, since T2DM patients being treated with metformin did not exhibit any susceptibility for BP. Furthermore, metformin has been used for several decades without any noted induction of BP [76].

Though BP typically has a severe clinical presentation, many cases can be completely resolved with topical or systemic steroids and discontinuation of immune checkpoint inhibitors, while other cases may require rituximab or omalizumab. In the above-reported cases, patients showed successful response and recovery with steroid treatment. This confirms that all the patients in this study exhibited a strong association between BP and ICI-induced autoimmunity. With regard to the WHO-UMC Causality Categories, all our patients may be classified as “certain” due to the fact that there was no other medical intervention that could possibly be responsible for this, i.e., any other causes could confidently be ruled out [77].

In our cases, patients ranged from 47 to 69-years-old. BP most commonly affects people over 60-years-old, as seen in two of our patients; however, it may also appear in younger populations as well, including children. Our patients had IgG antibodies along the basement membrane, as well as C3 and fibrinogen deposits along the membrane. Indirect immunofluorescence was negative in all three cases. This is the first published case of brain pinoblastoma treated with nivolumab presenting with BP as an adverse event. This is an unusual case of pinoblastoma in a patient who is not very young. This is also the first reported case of BP developing as an adverse reaction of pembrolizumab used in the treatment of UC.

Further research should investigate the exact mechanisms of BP as an ICI adverse event, as well as the reasons why some immunotherapies have shorter times between medication and BP onset, while others have longer times between medication and onset.

## 7. Conclusions

Immune checkpoint inhibitors cause many immune-related side effects, most commonly cutaneous toxicities. Bullous pemphigoid, a rare blistering adverse effect occurs in few patients but often has a severe clinical presentation. Due to the considerable morbidity and mortality, it is important that clinicians are aware of common symptoms, lab results, and expected onsets of BP following initiation of immune checkpoint inhibitors. Treatment typically includes steroids and, in some cases, cessation of immunotherapy.

## Figures and Tables

**Table 1:** Summary of cases.

Case	IgG	IgA	IgM	C3	Fibrinogen	Indirect Immunofluorescence	Pathology Findings of Skin Biopsy
1	+3/+4 linear, continuous deposits along the basement membrane	negative	+1 linear, continuous deposits along the basement membrane.	+4 linear, continuous deposits along the basement membrane.	+3 linear, continuous deposits along the basement membrane.	Negative	Infiltrate composed of mononuclear cells, numerous eosinophils, and sparse neutrophils in upper dermis, consistent with subepidermal blistering disease.
2	+3/+4 linear, continuous deposits along the basement membrane	negative	+1 focal, continuous deposits along the basement membrane	+2 granular, continuous deposits along the basement membrane.	+2 linear, continuous deposits along the basement membrane.	Negative	Infiltrate composed of mononuclear cells, numerous eosinophils, and sparse neutrophils in upper dermis, consistent with subepidermal blistering disease.
3	+3/+4 linear, continuous deposits along the basement membrane	positive	negative	.+4 linear, continuous deposits along the basement membrane.	+3 linear, continuous deposits along the basement membrane.	Negative	Infiltrate composed of mononuclear cells, numerous eosinophils, and sparse neutrophils in upper dermis, consistent with subepidermal blistering disease.

**Table 2:** Immune Checkpoint Inhibitors, Indications, and Side Effects.

Drug	First Approved	Cancers Approved for Treatment	Most Common Side Effects
Ipilimumab	2011	Melanoma, RCC, CRC, HCC, NSCLC	Fatigue, diarrhea, pruritis, rash, colitis
Pembrolizumab	2014	Melanoma, lung cancer, SCC, lymphomas, urothelial carcinoma, cancers high in MSI, MMR-deficient cancers, gastric cancers, esophageal cancers, cervical cancers, HCC, Merkel cell cancer, RCC, endometrial carcinoma, high tumor mutational burden- cancer, triple-negative breast cancer	Fatigue, musculoskeletal pain, decreased appetite, diarrhea, rash, fever, cough, constipation, nausea, abdominal pain, pruritis
Nivolumab	2014	Melanoma, NSCLC, malignant pleural mesothelioma, RCC, classic Hodgkin lymphoma, HNSCC, urothelial carcinoma, CRC, HCC, esophageal squamous cell carcinoma	Fatigue, rash, pruritis, diarrhea
Atezolizumab	2016	Urothelial carcinoma, NSCLC, triple-negative breast cancer, SCLC, HCC, melanoma	Fatigue, nausea, vomiting, cough, dyspnea, decreased appetite, alopecia, constipation or diarrhea, headache, rash
Durvalumab	2017	Urothelial carcinoma and NSCLC	Fatigue, constipation, UTIs, edema, pneumonitis, dyspnea, rash, cough, nausea
Cemiplimab	2018	cSCC, BCC and NSCLC	Pneumonitis, colitis, hepatitis, endocrinopathies, dermatologic reactions, musculoskeletal pain, fatigue, rash, and diarrhea

RCC, renal cell carcinoma; CRC, colorectal cancer; HCC, hepatocellular carcinoma, NSCLC, non-small-cell lung cancer; SCC, squamous cell carcinoma; MSI, microsatellite instability; MMR, mismatch repair; HNSCC, head and neck squamous cell carcinoma; SCLC, small-cell lung cancer; UTIs, urinary tract infections; cSCC, cutaneous squamous cell carcinoma; BCC, basal cell carcinoma.

**Table 3:** Weeks until presentation of ICI-associated BP based on immunotherapy.

Immunotherapy	Average (Weeks) to Develop BP	Number of Studies on Which the Average Is Based
Durvalumab then Nivolumab	52	1
Nivolumab then Ipilimumab	50	1
Atezolizumab	49.5	2
Durvalumab and Tremelimumab	42	1
Pembrolizumab	35.3	14
Ipilimumab then Pembrolizumab	30.5	2
Pembrolizumab then Nivolumab	26	1
Ipilimumab then Nivolumab	24	1
Pembrolizumab and Ipilimumab	24	1
Ipilimumab and Nivolumab then Nivolumab	22	1
Nivolumab	19.8	24
Cemiplimab	9	1
Ipilimumab	2	1
Pembrolizumab then Ipilimumab	1	2

**Table 4:** ICI-associated dermatotoxicities and their characteristics.

Immune-Related Adverse Effect	Most Common Symptoms
Pruritus	Itch with or without rash
Morbilliform exanthem	Transient and coalescing pink macules and papules
Vitiligo-like depigmentation	Loss of skin pigmentation, halo nevi
Lichenoid dermatitis	Pruritic, violaceous papules/plaques, may involve mucosal surfaces
Bullous pemphigoid	Tense vesicles/bullae, erosions, urticarial plaques, pruritus
Stevens–Johnson Syndrome/Toxic Epidermal Necrolysis (SJS/TEN)	Dark patches on skin and mucous membranes, epidermal necrosis and sloughing
Acute Generalized Exanthematous Pustulosis (AGEP)	Erythematous and edematous plaques covered in pustules, fever, facial edema, may involve mucosal surfaces
Drug Reaction with Eosinophilia and Systemic Symptoms (DRESS)	Morbilliform rash that may be indurated or purpuric, fever, facial edema, lymphadenopathy, end-organ dysfunction

## Data Availability

Data are contained within the article or are available from the authors upon reasonable request.

## Abbreviations

ICI	Immune checkpoint inhibitor
irAE	Immune-related adverse event
BP	Bullous Pemphigoid
SCARS	Severe cutaneous adverse reactions
SJS/TEN	Stevens–Johnson Syndrome/Toxic Epidermal Necrolysis
AGEP	Acute Generalized Exanthematous Pustulosis
DRESS	Drug Reaction with Eosinophilia and Systemic Syndromes
DIF	Direct immunofluorescence
IIF	Indirect immunofluorescence
EBA	Epidermolysis bullosa acquisita

## Appendix A

**Table A1:** Weeks until presentation of ICI-associated BP based on immunotherapy.

Sex (Male/Female)	Age (Years)	Primary Tumor	Immunotherapy	Clinical Presentation	Time to Develop BP (Weeks)	Treatment	Refs.
Male	75	Melanoma	Pembrolizumab	Cutaneous BP	22	Oral steroids	[78]
Male	63	Tongue SCC	Nivolumab	Oral and cutaneous BP	8	Topical/oral steroids	[79]
Male	68	Melanoma	Pembrolizumab	Cutaneous BP	16	Topical/oral steroids	[79]
Female	74	Urothelial Ca.	Nivolumab	Cutaneous BP	18	IV/oral steroids	[79]
Female	73	NSCLC	Nivolumab	Cutaneous BP	6	Oral and topical steroids and nicotinamide	[79]
Male	68	Melanoma	Nivolumab	Cutaneous BP	3	IV/oral steroids	[79]
Male	72	Melanoma	Ipilimumab	Severe BP exacerbation	2	Oral steroids	[80]
Female	77	Lung cancer	Nivolumab	Cutaneous BP	6	Oral steroids and omalizumab	[81]
Male	63	Melanoma	Pembrolizumab	Cutaneous BP	84	Topical and oral steroids	[65]
Male	80	Melanoma	Nivolumab after Ipilimumab	Cutaneous BP	24	Topical and oral steroids	[47]
Female	78	Melanoma	Nivolumab after Durvalumab	Cutaneous BP	52	Topical steroids	[47]
Male	85	SCC of lung	Nivolumab	Cutaneous BP	18	Oral and topical steroids	[47]
Female	78	Melanoma	Pembrolizumab after Ipilimumab	Cutaneous BP	32	Oral and topical steroids	[47]
Male	68	Melanoma	Pembrolizumab	Oral and cutaneous BP	78	Topical steroid	[62]
Male	72	Melanoma	Pembrolizumab	Oral and cutaneous BP	18	Oral steroid and methotrexate	[62]
Male	75	Melanoma	Pembrolizumab	Recurrent BP	4	Oral steroid	[62]
Male	70	Melanoma	Nivolumab after Pembrolizumab	Localized BP	26	Monitoring	[82]
Male	80	Lung	Nivolumab	Oral and cutaneous BP	80	IV Methylpred+ rituximab	[67]
Male	42	Melanoma	Pembrolizumab	Prolonged BP	44	Oral and topical steroids	[83]
Male	60	Renal cell Ca.	Nivolumab	Cutaneous BP	12	Oral and topical steroids	[84]
Male	73	Melanoma	Pembrolizumab	Cutaneous BP	24	Niacinamide	[85]
Male	90	Melanoma	Nivolumab	Cutaneous BP	12	Oral and topical steroids	[85]
Female	56	Melanoma	Pembrolizumab and Ipilimumab	Cutaneous BP	24	IV and oral steroids and methotrexate	[86]
Male	65	Melanoma	Pembrolizumab	Cutaneous BP	51	Oral steroids	[87]
Male	80	Skin SCC	Nivolumab	Cutaneous BP	30	Oral steroids and dapsone	[88]
Male	85	Melanoma	Nivolumab	Cutaneous BP	8	Topical steroids	[88]
Female	77	Urothelial Ca.	Atezolizumab	Cutaneous BP	21	Topical/oral steroids and omalizumab, methotrexate	[48]
Male	77	NSCLC	Nivolumab	Cutaneous BP	13	Topical/oral steroids	[48]
Female	77	NSCLC	Nivolumab	Cutaneous BP	3	Topical/oral steroids and omalizumab	[48]
Male	69	NSCLC	Nivolumab then Pembrolizumab	Cutaneous BP	4	Topical and oral steroids and nicotinamide	[48]
Male	68	Melanoma	Pembrolizumab then Ipilimumab and Nivolumab	Cutaneous BP	3	Topical and oral steroids and nicotinamide	[48]
Male	48	Renal cell Ca.	Nivolumab	Cutaneous BP	43	Oral steroids and nicotinamide	[48]
Female	61	NSCLC	Pembrolizumab	Cutaneous and oral BP	39	Topical and oral steroids	[48]
Female	83	Melanoma	Pembrolizumab	MMP	66	Doxycycline only	[89]
Male	64	Melanoma	Pembrolizumab	Cutaneous BP	12	Topical and oral steroids	[90]
Male	71	Melanoma	Ipilimumab then Pembrolizumab	Cutaneous BP	29	Topical and oral steroids	[90]
Female	70	NSCLC	Nivolumab	Cutaneous BP	7	Topical and oral steroids and niacinamide	[91]
Male	72	Melanoma	Ipilimumab after pembrolizumab	BP	1	Oral and topical steroid	[58]
Male	60	NSCLC	Nivolumab	BP	52	Oral steroids	[92]
Male	35	Melanoma	Nivolumab then Ipilimumab	BP	50	Topical steroids	[59]
Male	62	Merkel cell Ca.	Pembrolizumab	MMP	13	Topical steroids	[93]
Male	64	Melanoma	Nivolumab	Oral and cutaneous BP	18	Topical steroids	[94]
Male	62	Renal cell Ca.	Nivolumab	BP on higher dose immunotherapy	1	Oral steroids	[95]
Female	69	Melanoma	Nivolumab	Cutaneous BP	11	Topical/oral steroids and dapsone	[96]
Female	70	Melanoma	Nivolumab	MMP	12	Topical steroids	[97]
Female	47	Ovarian Ca.	Pembrolizumab	Severe MMP	3	Topical and oral steroids	[98]
Male	74	Lung	Nivolumab	Oral and cutaneous BP	50	Oral steroid	[99]
Male	82	Penile SCC	Atezolizumab	Photodistributed BP	52	Oral steroids	[100]
Male	87	Urothelial cell Ca.	Atezolizumab	Cutaneous BP	77	Topical steroids, doxycycline and niacinamide	[101]
Male	73	Renal cell Ca.	Nivolumab	Cutaneous BP	52	IV and oral steroids	[102]
Female	69	NSCLC	Durvalumab and Tremelimumab	BP	42	Oral steroids	[103]
Male	68	Cutaneous SCC	Cemiplimab	BP	9	Oral steroids and rituximab	[104]
Male	66	Renal cell Ca.	Nivolumab	Cutaneous BP and MMP	36	IV and oral steroids	[105]
Male	87	Melanoma	Nivolumab	BP	8	Topical and oral steroids	[106]
Male	72	melanoma	Pembrolizumab then Ipilimumab	Oral and cutaneous BP	1	Topical and oral steroids	[39]
Male	47	Brain pinealoblastoma	Nivolumab	Cutaneous BP	8	Topical and oral steroids	Current work
Male	69	Renal cell Ca.	Ipilimumab and Nivolumab then Nivolumab	Cutaneous BP	22	Topical and oral steroids	Current work
Male	69	Urothelial cell Ca.	pembrolizumab	Cutaneous BP	34	IV and oral steroids and topical	Current work

SCC: squamous cell cancer; Ca.: carcinoma; NSCLC: non-small-cell lung cancer; MMP: mucous membrane pemphigoid; BP: bullous pemphigoid.
